# A practical and nuanced framework for entity linking evaluation

**DOI:** 10.1186/s13326-025-00339-0

**Published:** 2026-02-12

**Authors:** Fuqi Xu, Goran Nenadic, Robert Stevens

**Affiliations:** Department of Computer Science, University of Manchester, Kilburn Building, Oxford Road, Manchester, M13 9PL UK

**Keywords:** Entity linking, Evaluation, Hierarchical vocabulary, Benchmarking

## Abstract

**Background:**

Entity linking maps textual mentions with entities in vocabularies. While accuracy is the primary metric for entity linking evaluation, it fails to capture the complexity of model behaviour.

**Results:**

We propose an entity linking evaluation framework that clarifies the target objects of metrics calculation, incorporates term hierarchy, generates performance profiles, and summarises them as model characteristics. The framework emphasises hierarchical vocabulary structures, shifting the focus from text-level label matching to semantic comparison between concepts. We illustrate the competence and utility of this framework through a case study on disease entity linking.

**Conclusion:**

Our results highlight the importance of aligning evaluation metrics with application-specific requirements, and provide structured hierarchical error analysis for entity linking, paving the way for more nuanced and practical assessments of entity linking systems.

## Background

Entity linking (EL), also known as entity normalisation or disambiguation, maps mentions, i.e., specific occurrences of text within documents, to entities in established vocabularies [13]. EL typically follows named entity recognition (NER), where NER models identify relevant spans and EL models link these spans to entities in structured vocabularies [5]. EL systems learn semantic patterns from training datasets and are evaluated on test datasets using metrics such as accuracy, i.e., the percentage of mention-entity pairs that precisely match those in the test data.

Multiple approaches have been used to evaluate entity linking, including standard metrics such as F1, macro- and micro-accuracy, and the least common ancestor F-score, which accounts for partial correctness [15]. Although accuracy remains the primary metric for EL benchmarking, model comparison, and selection [5, 7, 13], a single numeric score cannot fully represent a model’s behaviour [19]. First, models with similar overall scores may exhibit distinct linking patterns. For example, some models may prioritise recall while others emphasise precision [5, 7, 13],^1^ or a model may achieve strong overall performance while consistently mislinking entities in specific domains. As a result, marginal differences in scores may not reflect meaningful distinctions in model behaviour. Second, performance can vary for the same model on the same dataset depending on the experimental setup.^1^

Effective biomedical entity linking evaluation should account for hierarchical vocabularies, where entities are organised across multiple levels of abstraction. Standard accuracy metrics do not capture whether a model preserves these relationships, which are often crucial for downstream reasoning and task-specific decisions. In practice, the appropriate level of specificity varies by use case. For example, the mention “Granny Smith” may need to be linked to a specific cultivar in an agricultural database, where fine-grained distinctions matter, whereas in a retail system, it may be linked to a broader category such as *Fruit* for inventory management. These domain-specific preferences reflect differences in how hierarchical structures are interpreted and used. To address this, entity linking should be evaluated from multiple perspectives, with metrics selected to suit the goals of each application. Incorporating these considerations into model evaluation allows systems to better align with real-world needs.

This paper addresses key challenges in entity linking evaluation by introducing a hierarchy-aware assessment framework, refining generalisation analysis through representative evaluation sets, and supporting task-specific metric selection.

### Scope

A rigorous evaluation of entity linking should assess every stage of the process. This paper focuses on challenges in test dataset representativeness and performance assessment within hierarchical vocabularies. Key issues are discussed in this section, while proposed solutions are presented in the Results section.

### Challenge 1: Representativeness of the test dataset

Many EL models exhibit poor generalisability to real-world scenarios [10, 24] due to data drift and distributional change [8]. The representativeness of test datasets can affect a model’s generalisation and adaptivity [23]. One key issue is repetition, where performance metrics are skewed towards frequently occurring mentions rather than unique ones. For example, in the BC5CDR dataset, only 653 out of 4,363 disease mentions are unique, illustrating the prevalence of repetition. Furthermore, test datasets are often small, covering only 0.03% to 0.60% of the corresponding vocabularies.^2^ These limitations are further exacerbated by overlaps among training, development, and test sets, where many mentions or entities reappear. For instance, Garda et al. [7] found that novel mentions, those absent from the training and development sets, constitute only 4.06% to 19.79% of test sets in common benchmarking datasets. Our analysis of popular datasets^3^ similarly shows that model performance correlates with training-set coverage. Such overlaps and duplications can inflate metrics, as models may succeed due to overfitting rather than actual contextual resolution.

### Challenge 2: Consistent formats and processing strategies

Another issue refers to EL output formats, in which some entity linking models outputs recognised entities without exact text span mappings [22], making it difficult to separate NER outputs from entity linking results. Structured formats like PubTator [25] facilitate this separation by including start and end indices, selected text, and identified entities for each document. This allows EL evaluation to be performed using spans that exactly match gold-standard annotations, which is especially useful when mentions appear multiple times.

A consistent approach is also needed for edge cases, such as unlinked text spans, spans linked to multiple entities, or entities linked to a single multi-term expression [27].^4^ Without a standard dataset processing approach, cross-model comparisons are unreliable, compromising both model evaluation and selection.

### Challenge 3: Hierarchically arranged entities

In specialised domains such as biomedicine, entity linking typically maps to structured vocabularies, such as MeSH [18], or the Open Biological and Biomedical Ontologies (OBO) [21], where hierarchical relationships (e.g., *is-a*), organise concepts at varying levels of granularity. Current accuracy-based metrics classify a match solely based on whether it exactly matches the entity in the test dataset, ignoring the vocabulary’s hierarchical structure and variations in specificity among entities. A more informative evaluation should consider parent-child relationships and the broader semantic context of a mention [1, 3]; for example, in a hierarchical vocabulary, mapping text *“Granny Smith”* to entity *Fruit* (instead of *Apple*) may be contextually acceptable despite lacking specificity.

### Challenge 4: Hits and misses

In addition to evaluating performance on a specific dataset, a comprehensive assessment should test how well an EL model generalises to other datasets and real-world applications. Capturing both the strengths and weaknesses of a model across diverse evaluation settings offers a clearer picture of its robustness and applicability beyond the dataset it was trained on. This perspective also helps researchers and practitioners diagnose model failures and prioritise metrics that align with their specific objectives.

## Methods

We define entity linking as a function $f: \mathcal{M} \rightarrow \mathcal{O}$, which maps a set of textual mentions $\mathcal{M} = \{m_1, m_2, \dots, m_n\}$ to a set of entities within a vocabulary $\mathcal{O} = \{E_0, E_1, \dots, E_k\}$. For each mention *m*_*i*_, the function $f(m_i) = E_j$ assigns the corresponding entity *E*_*j*_ from $\mathcal{O}$. The evaluation of entity linking checks the relationship between target entities ${E_t}$ and the predicted entities *E*_*p*_.

We adopt terminology from the Web Ontology Language (OWL) recommendations [2] to develop the evaluation metrics. The distance between a target entity *E*_*t*_ and a predicted entity *E*_*p*_ is the shortest path connected by the *subclassOf* relationship. The *ancestor entities* of *E*_*t*_ are those with *E*_*t*_ as a direct or indirect subclass. Its *parent entity* has a distance of 1. Similarly, *descendant entities* are subentities of *E*_*t*_, with *child entities* being the immediate subentities. For orthogonal entities, their *lowest common ancestor* is the most granular entity containing both *E*_*t*_ and *E*_*p*_ as subsets. The *depth* of an entity is defined as the shortest distance to the root and represents its granularity [26].^5^

The proposed metrics were tested on two entity linking models, TaggerOne [16] and BioSyn [14] on the BC5CDR disease dataset [17]. TaggerOne is an early model that employs semi-Markov classifiers and learns from lexical information, while BioSyn is a transformer-based model designed for synonym resolution. These models were selected for their distinct mechanisms in entity linking, potentially yielding different performance behaviours.^6^ In this evaluation, we focused on the performance of the entity linking set, isolating it from the impact of NER. To ensure fair comparison, we selected only mentions with exact spans matching the test set where possible.^7^ Terms with multiple labels introduce additional complexity. When a single text span is linked to multiple entities in the gold standard, these are treated as separate pairs in the overall calculations.

## Results

### The target of an evaluation

We propose four target assessment sets to emphasise two key evaluation aspects: novel mentions, entities absent from training and development data, and the impact of repeated mention-entity pairs.

- **Global Set**: The entire test set.
- **Global Unique Set**: The mention-entity pairs in the test set with repetitions removed.
- **Novel Set**: A set of mention-entity pairs that only appear in the test dataset, and are not present in the training or development sets.
- **Novel Unique Set**: A set of novel mention-entity pairs with repetitions removed.

Each evaluation set supports an independent assessment of performance metrics. Take *accuracy* as an example: *Global Accuracy* measures the percentage of all mention-entity pairs that exactly match those in the gold standard dataset; *Global Unique Accuracy* considers only distinct mention-entity pairs by removing duplicates; *Novel Accuracy* evaluates the percentage of correctly linked mention-entity pairs that are absent from the training or development sets but present in the gold standard; and *Novel Unique Accuracy* measures the accuracy of distinct mention-entity pairs that meet the same criteria.

The selection of an appropriate evaluation set depends on the application scenario. Current EL experiments mainly use the *Global set* for overall evaluation, whereas the *Novel Unique set* is particularly useful for evaluating a model’s ability to generalise to unseen data. When a training dataset is unavailable, results on the *Novel set* are identical to those on the *Global set*.

The choice between the *Global Set* and *Global Unique Set* depends on how well the test corpus reflects the characteristics of datasets in the target application domain. Both the *Novel Set* and *Novel Unique Set* are especially valuable when working with extensive vocabularies or small test datasets.

### Performance profile for hierarchical entity linking

The "parent-child" relationship in hierarchical vocabularies can lead to mismatches in entity linking, which may vary in severity and correctness. To address this, we analyse the features of each match by considering the location, relationship, and hierarchical distance between the predicted entity and the target entity. These features are then summarised to construct a detailed performance profile for each model across different datasets.

### Match types

Four types of matches are defined based on the logical relationship between the target class *E*_*t*_ and the predicted entity *E*_*p*_. A match is *correct* if the logical relationship between *E*_*t*_ and *E*_*p*_ holds. The correctness doesn’t imply the match is optimal or useful.

- **Exact match**, $\mathcal{E}$: The predicted entity is identical to the target entity, $E_t = E_p$, denoted as $\leftrightarrow$.
- **Overspecific match**, $\mathcal{O}$: The predicted entity is a subset of the target entity, $E_t \subset E_p$, denoted as $\rightarrow$. An overspecific match may still be correct if it is consistent with the actual concept.
- **Underspecific match**, $\mathcal{U}$: The predicted entity is a superset of the target entity, $E_p \subset E_t$, denoted as $\leftarrow$. Underspecific matches, although lacking specificity, are always correct since they are mapped to a more general parent entity.
- **Orthogonal match**, $\mathcal{T}$: The predicted entity is neither a subset nor a superset of the target entity. $\neg (E_t \subseteq E_p) \land \neg (E_p \subseteq E_t)$, such as sibling entities, denoted as $\perp$.

### Match distance

***Match Distance*** quantifies the degree of mismatch by measuring how far the predicted entity is from the target entity. For orthogonal matches, the distance equals the sum of distances from each entity to their lowest common ancestor. Within the same match type, greater distances indicate increased divergence. A correct match has a distance of 0, while a mapping to a direct parent or child has a distance of 1.

1 $$ \mathrm{Distance}(E_t, E_p) = \begin{cases} \mathrm{Dis}(E_t, E_p), & \text{if } E_t \cap E_p \neq \emptyset, \\ \begin{aligned}&|\mathrm{Dis}(E_t, E_x)| \cr&\quad+ |\mathrm{Dis}(E_p, E_x)|,\end{aligned} & \text{if } E_t \cap E_p = \emptyset. \end{cases} $$

where $\mathrm{Dis}(E_t, E_p)$ is the distance via the shortest path using the *SubClassOf* relationship within the vocabulary, and *E*_*x*_ is the lowest common ancestor of *E*_*t*_ and *E*_*p*_.

### Location of match

The position of a match captures both its position within the hierarchical structure, specifying the branch it belongs to, and its depth. Errors at closer to the root of the vocabulary, which correspond to making mistakes at broader categories, have a greater impact than those at lower levels. To account for this, we include *Location*, the relative depth of the target entity within its branch, for each entity-mention pair. This metric captures the severity of mismatches by reflecting variations in informativeness and semantic granularity across hierarchical levels.

2 $$ \mathrm{Loc}(E_t) = \frac{\min \mathrm{Dis}(E_t, \mathrm{root})}{\min \mathrm{Dis}(\mathrm{root}, \mathrm{leaf})} $$

where $\mathrm{Dis}(E_t, \mathrm{root})$ is the distance between *E*_*t*_ and the root, and $\min \mathrm{Dis}(\mathrm{root}, \mathrm{leave})$ is the distance from the root to the nearest leaf.

### Performance profile

We construct a ***performance profile*** to evaluate model performance on specific datasets, where each row *P*_*i*_ in Profile *P* corresponds to a mention-entity pair with the following properties:

3 $$ P = \begin{pmatrix} \mathrm{Type}_1 & \mathrm{Distance}_1 & \mathrm{Loc}_1 \\ \mathrm{Type}_2 & \mathrm{Distance}_2 & \mathrm{Loc}_2 \\ \vdots & \vdots & \vdots \\ \mathrm{Type}_n & \mathrm{Distance}_n & \mathrm{Loc}_n \\ \end{pmatrix} $$

where:

- $\mathrm{Type}_i$ is the match type of the *i*-th pair.
- $\mathrm{Distance}_i$ is the matching distance of the *i*-th pair, defined as the distance between the target entity and the predicted entity.
- $\mathrm{Loc}_i$ is the match location of the *i*-th pair, defined as the relative depth of the target entity in the vocabulary hierarchy.

**Fig. 1:**
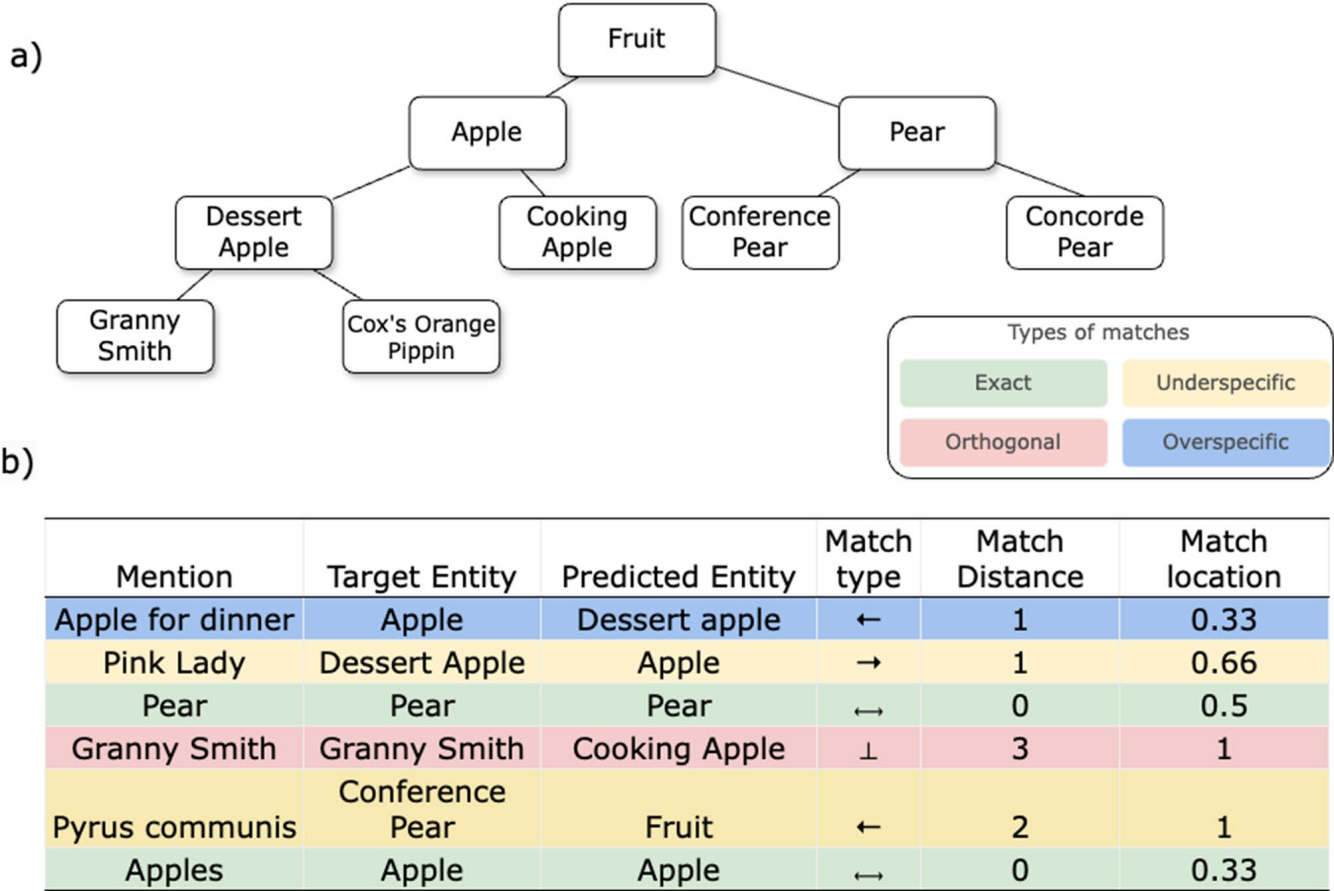
Entity linking performance profile for the example fruit vocabulary and sample dataset. **a**. Hierarchical structure of an example fruit vocabulary. **b**. Sample dataset and its entity linking performance profile, coloured by match types

Figure 1 illustrates the performance profile. Traditional entity linking assessment considers only *Exact matches*. In contrast, we further categorise matches into various mismatch types and represent these using a heatmap (Fig. 1b), capturing both types and degrees of mismatches. In this example, mapping “apple for dinner” to *dessert apple* is overspecific. While this may be valid, since a dessert apple is a type of apple, further context from the article could clarify whether this finer classification is appropriate. Conversely, linking “Pyrus communis” to *Fruit* instead of *Conference pear* is correct, though some specificity is lost.

### Model characteristics of hierarchical entity linking

The performance profile provides a comprehensive mapping of every mention-entity pair in the dataset, enabling detailed analysis. To describe a model’s features and support high-level comparisons, we summarise this profile into model characteristics that reflect the model’s tendencies in linking entities.

For the entire dataset, we introduce ***Coverage*** and ***Specificity***, alongside accuracy, as key metrics to characterise model performance. ***Coverage*** measures the proportion of entities accurately annotated, allowing broader, superclass matches (e.g., mapping “Apple” to *Fruit*) to increase coverage at the expense of specificity. In contrast, ***Specificity*** reflects the granularity of a predicted entity within the vocabulary. Overspecified matches (e.g., predicting *Pink Lady* instead of the target *Apple*) may be deemed correct or incorrect depending on the context and use case.

Let $\mathcal{D}$ be the set of all instances in the entity linking dataset and $\mathcal{X}$ be a specific type of match where $\mathcal{X} \in \{\mathcal{E}, \mathcal{O}, \mathcal{U}, \mathcal{T}\}$, and $N_{\mathcal{X}} = \left| \{x \in \mathcal{D} \mid x \text{is of type } \mathcal{X} \} \right|$ then:

4 $$\mathrm{Accuracy} = \frac{N_E}{N_E + N_\mathcal{O} + N_\mathcal{U} + N_P}$$

5 $$\mathrm{Specificity} = \frac{N_E+N_\mathcal{O}}{N_E + N_\mathcal{O} + N_\mathcal{U} + N_P}$$

6 $$\mathrm{Coverage} = \frac{N_E+N_\mathcal{U}}{N_E + N_\mathcal{O} + N_\mathcal{U} + N_P}$$

We also use ***Braveness***, ***Cautiousness*** and ***Orthogonality*** to measure cases where the model fails to predict the target entity.

7 $$\mathrm{Braveness} = \frac{N_\mathcal{O}}{N_\mathcal{O}+N_P+N_\mathcal{U}}$$

8 $$\mathrm{Cautiousness} = \frac{N_\mathcal{U}}{N_\mathcal{O}+N_P+N_\mathcal{U}}$$

9 $$\mathrm{Orthogonality} = \frac{N_\mathcal{P}}{N_\mathcal{O}+N_P+N_\mathcal{U}}$$

***Braveness*** reflects a model’s tendency to predict more specific entities, while ***Cautiousness*** captures its inclination to generalise predictions to higher-level categories, trading specificity for coverage. ***Orthogonality*** reflects the degree of orthogonal matches in predictions, representing mismatches that are always incorrect. While a brave or cautious mismatch can be accepted depending on the application, orthogonal mismatches should be minimised.

## Case studies: Performance comparison of two models in disease entity linking

### Accuracy assessment

We calculated the accuracy for TaggerOne and BioSyn across different BC5CDR disease subsets (See Table 1). Both models experienced a substantial performance drop (10.39%-14.24%) on the novel unique set compared to their global counterparts. The performance difference between the two models on the novel unique dataset is minimal (0.09%). For both models, the removal of repetition negatively impacts accuracy, with the global set being more affected than the novel dataset. TaggerOne has a larger drop (4.51%) in the global set.

**Table 1:** Accuracy of BioSyn and TaggerOne on the BC5CDR disease dataset

Metric	BioSyn	TaggerOne
Global accuracy	90.43%	95.27%
Global unique accuracy	86.82%	90.76%
Novel accuracy	80.53%	81.13%
Novel unique accuracy	80.04%	81.03%

### Performance profiles

**Fig. 2:**
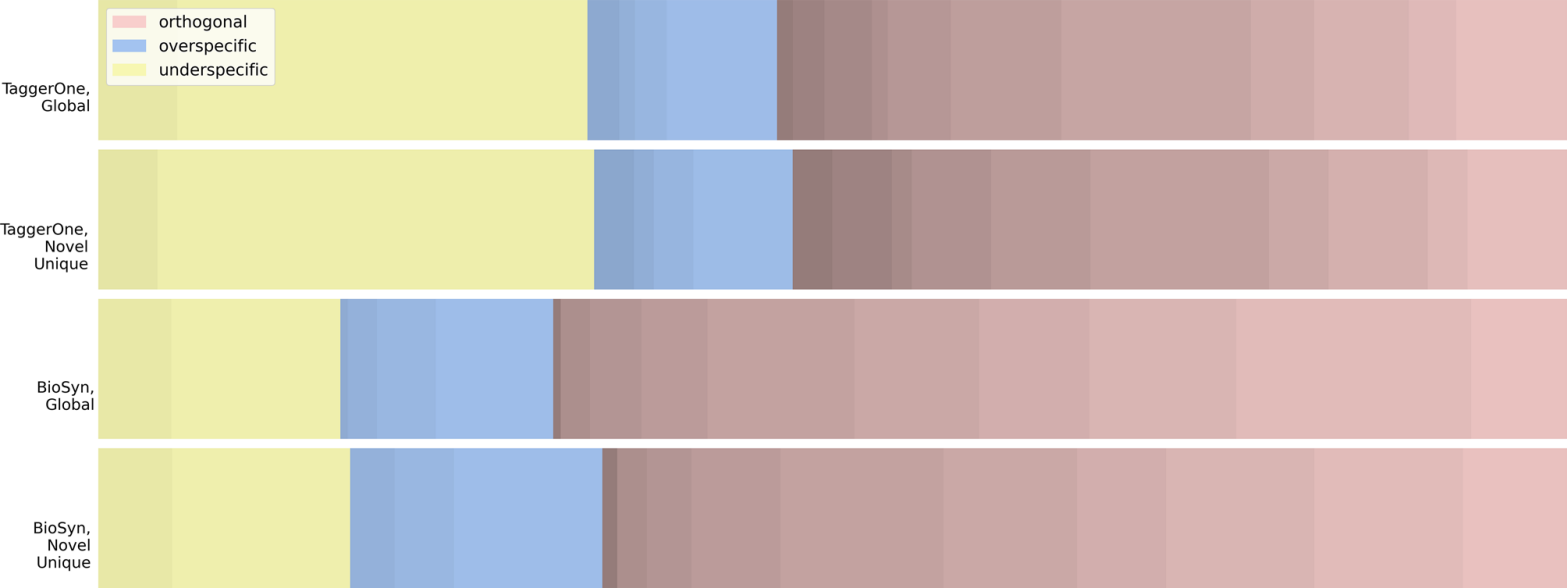
Performance profiles of TaggerOne and BioSyn (mismatches only). Error analysis highlights mismatched predictions. Darker shades indicate larger match distances

Although both models achieve similar overall accuracy, they display distinct performance patterns. Fig. 2 visualises the performance profiles of TaggerOne and BioSyn on the BC5CDR disease dataset, showing results for both the global set and the novel unique set. Over 50% of mismatches in both models are classified as orthogonal matches, suggesting that the models frequently fail to identify the correct entity branch.

TaggerOne produces approximately three times as many underspecific matches as overspecific ones, and shows 12.47–16.61% more underspecific matches than BioSyn within the mismatch set. A higher proportion of underspecific matches implies that TaggerOne is better at identifying correct ancestor entities, making its errors comparatively more acceptable.

BioSyn demonstrates a consistent error profile across both the global dataset and the novel unique set. In contrast, TaggerOne’s performance profiles vary between sets, with the novel unique set containing 8.39% fewer orthogonal matches. This indicates that, while both models achieve similar accuracy on the novel unique set, TaggerOne is more effective at avoiding mismatches with entities that are further apart. However, when TaggerOne does make orthogonal errors, the average distance of such mismatches is greater, indicating a higher degree of divergence from the target entity.

**Fig. 3:**
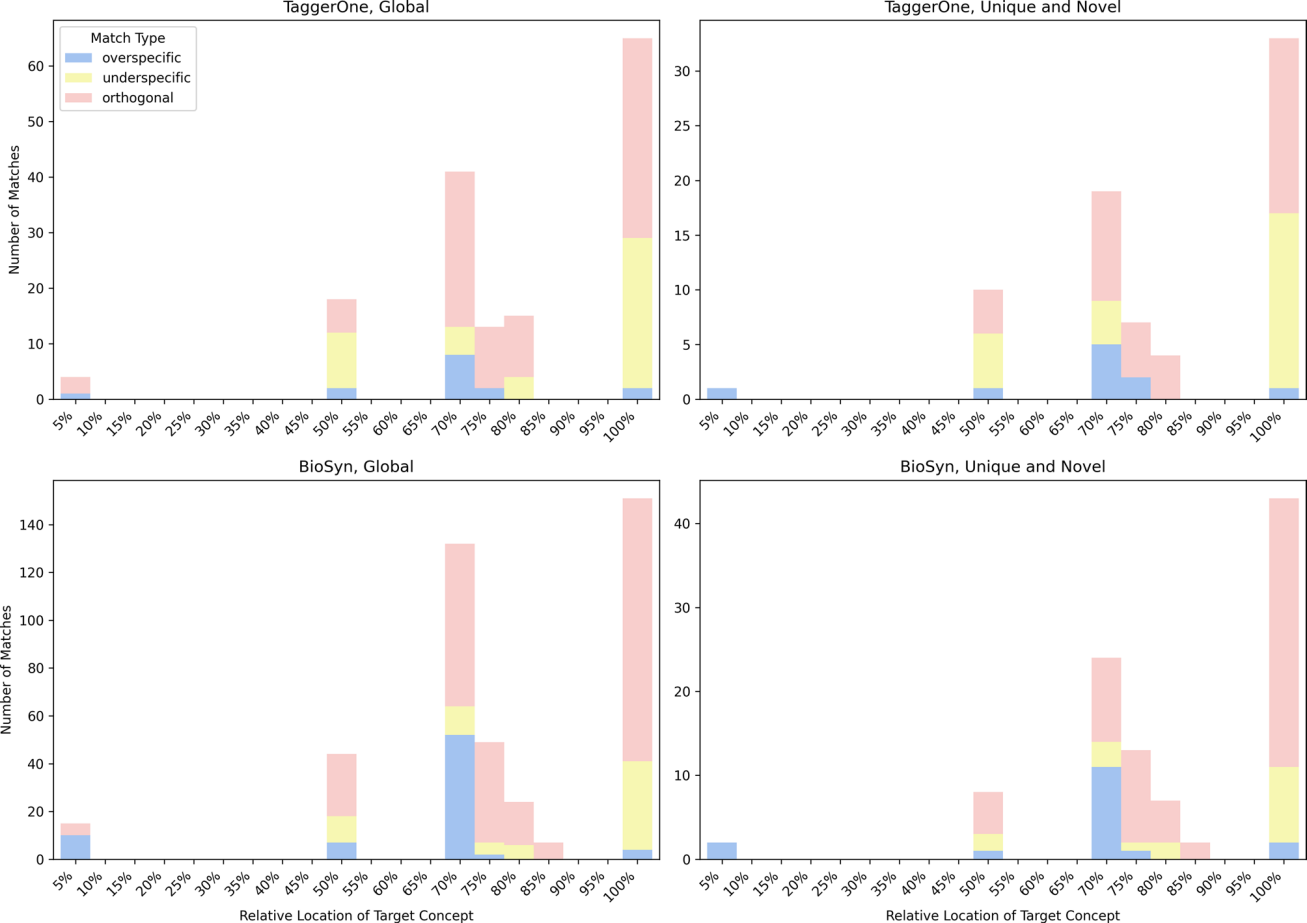
Model Performance by locations (mismatches only). The relative location refers to the position of the target entity within the shortest path of its corresponding branch in the vocabulary. A relative location of 1 indicates that the target entity is the most specific (i.e., leaf-level) node in its branch

Mismatch location analysis provides further insights into the models’ performance (Fig. 3). Nearly half of all mismatches occur at the leaf level. BioSyn shows a higher prevalence of overspecific matches at the mid-level (relative location 70%), while orthogonal mismatches dominate at the leaf level. Notably, BioSyn’s mismatch patterns remain consistent between the global set and the novel unique set.

In contrast, TaggerOne exhibits different behaviour in the novel unique set compared to the global set. In the novel unique set, underspecific matches are concentrated at the mid-level (relative location 50–70%), reflecting an overcautious approach that avoids linking to generic entities. At the leaf level, underspecific matches occur at a comparable rate to orthogonal matches, indicating that this cautious strategy effectively mitigates errors at finer levels of granularity.

### Characteristics of both models

Table 2 compares the characteristics of TaggerOne and BioSyn. TaggerOne achieves broader coverage across both global and unique novel sets, demonstrating its effectiveness in capturing a wider range of mentions. In contrast, BioSyn exhibits greater predictive braveness, particularly in the global set. TaggerOne, by comparison, demonstrates a more cautious prediction strategy, especially in the novel unique set. Despite similar levels of orthogonality, BioSyn generates a greater proportion of orthogonal errors in the unique novel set, with most errors occurring at leaf nodes. These findings highlight a model characteristics contrast: TaggerOne prioritises coverage and caution, while BioSyn makes brave overspecific predictions, with a higher risk of errors. These complementary characteristics support informed model selection based on specific application requirements, for example, prioritising coverage in low-recall scenarios or favouring risk-tolerant models when high precision is essential.

**Table 2:** Characteristics of the BioSyn and TaggerOne model based on their performance on the BC5CDR disease dataset

	Overall analysis			Error analysis		
	Accuracy	Coverage	Specificity	Braveness	Cautiousness	Orthogonality
BioSyn Global	90.43%	92.04%	92.13%	17.60%	16.82%	65.58%
BioSyn Unique Novel	80.04%	83.48%	83.48%	17.17%	17.17%	65.66%
TaggerOne Global	95.27%	96.67%	95.73%	9.61%	29.29%	61.10%
TaggerOne Unique Novel	81.02%	87.44%	83.59%	13.51%	33.78%	52.71%

### The competency of an entity linking model

We recommend evaluating models by aligning their competencies with specific use case requirements. Users can customise their selection of evaluation metrics. Below, we outline three example scenarios, summarised in Table 3.

**Table 3:** Metrics of interest based on entity linking scenarios. Metrics of interest are marked with “x”

Scenario	Match Type	Match Distance	Match Loc	Accuracy	Coverage	Specificity	Braveness	Cautiousness	Orthogonality
*Cancer branch*	E, U, O, S		x	x					
*Insurance reimbursement*	E, U		x	x	x			x	x
*Annotation recommendation*	E, U, O, S	x	x	x					

#### Scenario 1: Focusing on the position

When multiple vocabularies are integrated, evaluation performance can be biased if mappings outside the target subdomain of a particular vocabulary are included. For example, although the NCI Thesaurus (NCIt) [4] is widely used for cancer modelling, it also contains extensive non-cancer branches such as food and drug materials. In this scenario, while filtering could in principle be applied to retain only cancer concepts, a proper evaluation should emphasise where the mismatch occurs and annotation accuracy.

#### Scenario 2: Prioritising coverage

When mapping insurance claims to SNOMED CT [20], links where a term is mapped to underspecific concepts are tolerated considering the annotation difficulty and the size of the vocabulary [12]. In this context, unmapped diseases risk under-reimbursement, while incorrect mappings risk misclassification of reimbursement categories. Moreover, fine-grained disease subtypes have limited relevance for insurance purposes. Therefore, together with accuracy, cautiousness, and orthogonality should be prioritised.

#### Scenario 3: Finding the neighbourhood

When data curators use an entity linking model [11] to facilitate their manual annotation by pre-selecting several candidate entities. The EL models is supposed to provide candidates in the right area of the vocabulary for human experts to further select the most suitable. In this case, for models with comparable accuracy, those with shorter average distance to the target entity are better suited.

## Discussion

Our findings reflect a broader need for evaluation frameworks that go beyond surface-level accuracy to capture nuanced model behaviour. The proposed metrics and analysis techniques represent a practical step toward more meaningful and actionable model assessment in biomedical entity linking. By incorporating hierarchical information, our evaluation better aligns with the structured nature of biomedical vocabularies, offering deeper insight into the semantic appropriateness of model outputs. This is particularly valuable in ontology-driven domains, where distinctions between entity types often reflect complex biological relationships.

The correlation between a model’s performance and the percentage of novel entities in the test set partially explains why many models fail to perform well on real-world datasets. This highlights the importance of conducting evaluations not only on the global set but also on novel subsets.

Our proposed evaluation metrics enhance accuracy-based calculations by capturing nuances in model behaviour and considering entity relationships within hierarchical vocabularies. These metrics are versatile and can be applied to various hierarchical structures, including taxonomies, thesauri, and ontologies. They are compatible in entity-level macro accuracy based assessments and mention-level micro accuracy based assessments.

TaggerOne originally used F1 for document-level entity linking evaluation. Although the research community has shifted towards accuracy-based evaluations in recent years, F1 measures remain relevant, particularly for multi-word expressions. Our evaluation approaches, including target set selection, hierarchical relationships, can be seamlessly integrated with F1 measurements and document-level evaluations by focusing one representative subsets or taking the hierarchy into account, offering a more comprehensive assessment framework.

While it is conceivable that future work may propose composite scores to summarise multiple aspects of performance, our focus here is on clarifying the underlying dimensions so that such aggregations, if pursued, can be better informed. A simple metric can obscure a model’s specific strengths and weaknesses. Instead, aggregating multiple metrics and designing custom measures tailored to specific use cases is more effective. For instance, error-tolerant systems might prioritise the accuracy of middle-level terms, ignore overspecific terms, and penalise underspecific ones in an aggregated score. Conversely, high-stakes systems requiring minimal errors should emphasise reducing orthogonal and overspecific mismatches. Users are expected to fully analyse their usage requirements and clearly define the competency questions before selecting their evaluation sets. This tailored approach ensures that evaluation metrics align with the diverse requirements of various applications.

While we have identified key challenges in entity linking assessment, the problem remains complex. Factors such as the similarity between the test dataset and the application dataset, the breadth and coverage of the vocabulary, and its size all influence assessment outcomes.

## Conclusions

Our framework allows users to make informed model choices tailored to their objectives and provides deeper insights into model errors. These insights enable targeted improvements, enabling entity linking systems be optimised for practical applications. We advocate transitioning from accuracy-based evaluation to use-case-driven assessment in entity linking. Metrics should align with competency questions that reflect user-specific needs, enabling users to select models and construct customized metric combinations based on these criteria.

## Figures and Tables

**Fig. A1:**
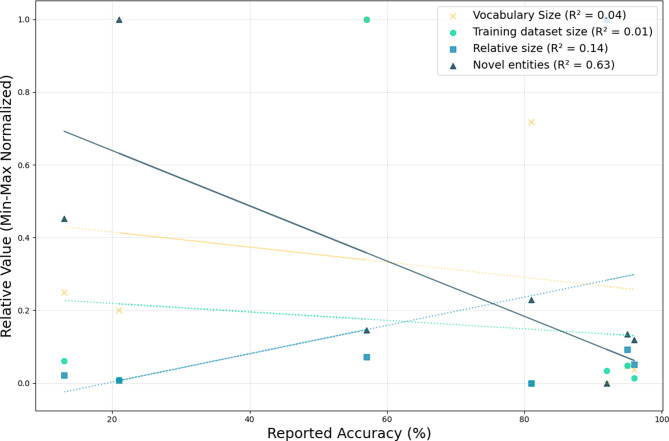
Corpus features and their impact on entity linking accuracy (BELB). *training dataset size*: number of annotations in the training/development set (if applicable). *relative size*: training dataset size divided by vocabulary size. *novel entities*: test entities absent from training/development sets. Solid trendlines indicate $R^2 > 0.5$, dashed otherwise

## Data Availability

No datasets were generated or analysed during the current study.

## Appendix 1: Features of common entity linking datasets

We selected the best-performing model for each corpus in the Biomedical Entity Linking Benchmark (BELB)  [7] and analyzed correlations between model accuracy and corpus features, including vocabulary size, training dataset size, the ratio of training data to vocabulary, and the proportion of novel entities in the test set. (See details in Table A1). Linear regression analysis revealed that only the proportion of entities first appearing in the test set significantly influenced model accuracy ($R^2 = 0.63$), whereas there is no evidence of vocabulary sizes or the size of training datasets contributing to the model accuracy ($R^2 < 0.1$) (See details in Fig. A1). This also confirms the need for a more representative test dataset.

**Table A1:** Features of common entity linking datasets and corresponding entity linking model performance

Entity linking dataset name	Corresponding vocabulary	Number of concepts in the vocabulary	Size of training dataset	Relative size of the training dataset (%)	Proportion of novel entity (%)	Accuracy (%)
BC5CDR-Disease	CTD Diseases	13,188	8,377	63.52	8.89	92
BC5CDR-Chemicals	CTD Chemicals	175,663	10,446	5.95	19.46	95
BioID	Cellosaurus	144,568	4,911	3.40	18.29	96
Linnaeus	NCBI Taxonomy	2,491,364	2,820	0.11	26.92	81
GNormPlus	NCBI Gene, GNormPlus Subset	703,858	4,218	0.60	87.59	21
MedMentions	UMLS 2017 AA	3,464,809	163,042	4.71	20.34	57
NLM-Gene	NCBI Gene, NLM-Gene Subset	873,015	12,634	1.45	44.52	13

Data collected from *Garda, et al. 2023*

Note that given confounding factors and the limited sample size, this conclusion highlights an issue for further investigation.

## Appendix 2: Summary of previous benchmarking experiments

Different models, when compared on the same benchmarking datasets, can have 10% differences in accuracy  [5, 13], while, in popular domains such as disease term linking, the differences narrow to approximately 3%  [7]. Nonetheless, even within a single model and the same dataset, accuracy can fluctuate between 3 and 6% depending on experimental setups. For instance, BioSyn [22] achieved 93.2% accuracy on the BioCreative V Chemical Disease Relation (BC5CDR) dataset  [17] but dropped to 88% on a variation of the same dataset in the Biomedical Entity Linking Benchmark (BELB), where all MeSH entities were converted to terms in the Comparative Toxicogenomics Database (CTD)  [4].

Datasets used in the BELB  [6] often have a small test dataset, comprising only 0.03% to 0.60% of the size of their corresponding vocabularies. (Exclduing datasets such as BC5CDR, NLM-Chem, and NCBI Disease are excluded here because they are evaluated against the CDT vocabulary instead of their original vocabulary.)

## Appendix 3: Formal definitions of key entities

- **Distance**:
  $$\mathrm{Distance}(E_g, E_c) = 2 \iff \exists E_p \ \big(E_c \subset E_p \subset E_g \big)$$
- **Class depth**:
  $$ d(C) = \min\left(\mathrm{distance}(C,R) \right) $$
  - where R represents the root term.
- **Ancestor entities**: $$E_0 \subseteq E_p $$
  - *E*_0_ is an ancestor class of *E*_*p*_ if *E*_0_ is a superclass (ancestor) of *E*_*p*_.
- **Parent entities**:
  $$ E_0 \subseteq E_p \land \mathrm{Distance}(E_g, E_c)=1 $$
  - *E*_*p*_ is the parent class of *E*_*c*_ if *E*_*c*_ is directly contained within *E*_*p*_, meaning there is no intermediate class.
- **Child/Descendant entities**: $E_c \subseteq E_0$
  - *E*_*c*_ is a child or descendant class of *E*_0_ if *E*_*c*_ is a subclass of *E*_0_, meaning it is contained within *E*_0_ directly or indirectly.
- **Sibling entities**:
  $$ E_s \iff \left(E_s \subseteq E_p \land E_0 \subseteq E_p \land E_s \neq E_0\right) $$
  - *E*_*s*_ is a sibling class of *E*_0_ if both *E*_*s*_ and *E*_0_ share the same parent class *E*_*p*_ but are distinct.
- **Lowest common ancestor entities**:
  $$ \mathrm{LCA}(E_a, E_b) = \max \{E_x \mid E_x \subseteq E_a \land E_x \subseteq E_b \} $$
  - The lowest common ancestor class between two entities *E*_*a*_ and *E*_*b*_ is the most specific class *E*_*x*_ that is an ancestor of both *E*_*a*_ and *E*_*b*_.
- **Orthogonal entities**:
  $$ E_a \parallel E_b \iff \left( E_a \cap E_b = \emptyset \right) $$
  - Entities *E*_*a*_ and *E*_*b*_ are orthogonal if they have no elements in common.

## Appendix 4: Dataset and model description

The BC5CDR dataset, consisting of 1,500 manually annotated PubMed documents linked to MeSH, serves as a key corpus for biomedical named entity recognition and entity linking tasks [18]. It includes disease and chemical subsets for entity linking. Within the disease subset, there are 4,423 annotated text spans, of which 4,363 are valid annotations. 63 of them valid text spans were annotated with multiple entities, separated by “$\vert$”. The training, development, and test sets comprise 4,149, 4,228, and 4,363 annotations, respectively. 8.89% of entities and 17.53% of mentions in the test set are absent from the training set  [7].

BioSyn leverages synonym information alongside morphological and semantic features to enhance entity linking. It achieves an accuracy of 93.2% on BC5CDR and 88% on the BELB test set  [22].

TaggerOne integrates named entity recognition and entity linking via a semi-Markov classifier. It employs TF-IDF weighting and normalisation techniques to generate vectors for lexicon entries, facilitating optimal entity matching. TaggerOne achieved a normalised F-score of 0.895 at the document level for the entire BC5CDR corpus [16], and an entity linking accuracy of 88.9% on the BC5CDR Disease dataset [22].

## Appendix 5: Metrics calculation

One of the challenges in the metrics calculation was to separate NER results with EL results, causing different formats used in TaggerOne and BioSyn outputs. This approach may result in a 2% performance variation from those reported, which we consider acceptable for this study.

TaggerOne outputs results in PubTator format [25], which although retaining necessary information, mixes named entity recognition with entity linking.

The BioSyn model uses a processed gold standard dataset containing 4,287 text spans, producing outputs that include text mentions, gold standard term IDs, and top candidate entities. It split and revised some multi-entity text spans to better align them with entities. For instance, in the original corpus, the mention of ‘Cerebral and pulmonary haemorrhage’ was annotated with two distinct entities in MeSH. Whereas, BioSyn divided the text into ‘Cerebral haemorrhage’ and ‘Pulmonary haemorrhage’, even though no explicit text spans in the source directly correspond to these entities. This revision complicates the identification of exact text span matches, as the revised spans do not fully align with the original context. Some text mentions were not split properly and were lost in corpus processing.

These differences in data formatting contribute to variations in calculated accuracy. To maintain compatibility with prior calculations while enabling meaningful comparisons, we adopted the steps listed below.

- **Separation of NER and EL results:** Due to BioSyn’s use of a revised gold standard that omits exact text span locations, we preserved its output as is. For TaggerOne, we only considered text spans that exactly matched annotated entities.
- **Handling multiple entities for a single text span:** Since BioSyn’s revised gold standard splits text spans with multiple entities into separate entities, we adopted a similar approach for TaggerOne results. Each entity within the same text span was treated as an individual annotation. For example, an annotation in the gold standard such as ‘Text Span A: MeSH_x $\vert$ MeSH_y’ was converted into two entities: “Text Span A: MeSH_x” and “Text Span A: MeSH_y”. The prediction is correct as long as the selected entity in the gold standards is in the prediction.
- **Handling multiple predictions for one gold standard.** We adopted a relaxed approach when matching multiple predictions to the gold standard. Such entities are only counted once and considered as correct as long as one prediction is correct. In cases where none of the predictions are correct, the first prediction is selected for downstream analysis.

The resulting BioSyn dataset contains 4476 annotated text spans, of which 4408 were successfully mapped to terms in an OWL-based representation of MeSH  [9] and used for downstream relationship analysis. They are also used in accuracy calculations.

For TaggerOne, we only kept linking results for text spans exactly the same as those in the gold standards, resulting in a significant decline of the comparable mention-entity pairs (3397 out of the 4364 pairs). 3301 of them were linked to terms in the MeSH file for subsequent analysis. This NER-EL separation step may inflate the entity-linking performance.

## Appendix 6: Multi-label impact

Accuracy is calculated as the number of *identical labels* divided by numbers in *the total set*. When text spans receive a single label for a distinct concept, the accuracy calculation is straightforward. However, the mapping between text spans, labels, and concepts is not always 1 to 1.

For instance, the text span “Cerebral and pulmonary haemorrhage” may be annotated with the MeSH labels “Cerebral haemorrhage,” “Pulmonary haemorrhage,” and “Hemorrhage”  [17]. Here, two distinct disease concepts are represented by three valid labels, with “Hemorrhage” being the superclass of the other terms. This shows the complexity of defining *identical labels* and *total set* when multiple labels map to concepts of mixed granularity.

In practice, *identical labels* can be interpreted as either “at least one label” being identical, or “all labels” matching. Similarly, *the total* may be calculated based on text spans or individual labels, with multiple labels often counted repeatedly.

## Abbreviations

BC5CDR: BioCreative V Chemical-Disease Relations
BELB: Biomedication entity linking benchmark
EL: Entity linking
MeSH: Medical subject heading
NER: Named entity recognition
OBO: Open biomedical ontology

## Footnotes

1 See details in Appendix 2: Summary of previous experiments.

2 See details in Appendix 2: Summary of previous experiments.

3 See Appendix 1: Features of common entity linking datasets.

4 See details in Appendix 6: Multi-label Impact.

5 Appendix 3: Formal Definitions provides formal definitions of these concepts.

6 Detailed descriptions of the dataset and models are provided in Appendix 4: Dataset and Model Description.

7 Further details on the separation of NER and EL, as well as the accuracy calculation and comparison, are provided in Appendix 5: Metrics Calculation.
